# Mapping staff perspectives towards the delivery of hospital care for children and young people with and without learning disabilities in England: a mixed methods national study

**DOI:** 10.1186/s12913-018-2970-8

**Published:** 2018-03-23

**Authors:** Kate Oulton, Faith Gibson, Lucinda Carr, Angela Hassiotis, Carey Jewitt, Charlotte Kenten, Jessica Russell, Mark Whiting, Irene Tuffrey-Wijne, Jo Wray

**Affiliations:** 1 Centre for Outcomes and Experience Research in Children’s Health, Illness and Disability (ORCHID), Great Ormond Street Hospital for Children NHS Foundation Trust, Level 4, Barclay House, 37 Queen Square, London, WC1N 3BH UK; 2 School of Health Sciences, Faculty of Health and Medical Sciences, University of Surrey, Guildford, Surrey GU2 7XH UK; 3 Great Ormond Street Hospital for Children NHS Foundation Trust, London, WC1N 3JH UK; 4 UCL Division of Psychiatry, 6th Floor, Maple House, 149 Tottenham Court Road, London, W1T 7NF UK; 5 UCL Knowledge Lab, Institute of Education, 23-29 Emerald Street, London, WC1N 3QS UK; 6 Health Research Building, University of Hertfordshire, College Lane Campus, Hatfield, Hertfordshire AL10 9AB UK; 7 Faculty of Health, Social Care and Education, Kingston University and St George’s University of London, 6th floor Hunter Wing, Cranmer Terrace, London, SW17 0RE UK

**Keywords:** Learning disability, Intellectual disability, Long-term conditions, Mixed methods, Health services research

## Abstract

**Background:**

Children and young people (CYP) with learning disabilities (LD) are a vulnerable population with increased risk of abuse and accidental injury and whose parents have reported concerns about the quality, safety and accessibility of their hospital care. The Care Quality Commission’s (CQC) view of best practice for this group of patients includes: access to senior LD nurse provision; a clearly visible flagging system for identifying them; the use of hospital passports; and defined communication strategies (Glasper, Comp Child Adolesc Nurs 40:63-67, 2017). What remains unclear is whether these recommendations are being applied and if so, what difference they are making. Furthermore, what we do not know is whether parental concerns of CYP with LD differ from parents of other children with long-term conditions. The aims of this study were to 1) describe the organisational context for healthcare delivery to CYP with LD and their families and 2) compare staff perceptions of their ability to identify the needs of CYP with and without LD and their families and provide high quality care to effectively meet these needs.

**Methods:**

Individual interviews (*n* = 65) and anonymised online survey (*n* = 2261) were conducted with hospital staff working with CYP in 15 children’s and 9 non-children’s hospitals in England. The majority of interviews were conducted over the telephone and recorded and transcribed verbatim. Health Research Authority was obtained and verbal or written consent for data collection was obtained from all interview participants.

**Results:**

The nature and extent of organisational policies, systems and practices in place within hospitals to support the care of CYP with LD differs across England and some uncertainty exists within and across hospitals as to what is currently available and accessed. Staff perceived that those with LD were included less, valued less, and less safe than CYP without LD. They also reported having less confidence, capability and capacity to meet the needs of this population compared to those without LD.

**Conclusion:**

Findings indicate inequality with regards the provision of high quality hospital care to children and young people with LD that meets their needs.

There is a pressing need to understand the impact this has on them and their families.

**Trial registration:**

The study has been registered on the NIHR CRN portfolio 20461 (Phase 1), 31336 (Phases 2-4).

**Electronic supplementary material:**

The online version of this article (10.1186/s12913-018-2970-8) contains supplementary material, which is available to authorized users.

## Background

In 2007 the UK learning disability charity Mencap published Death by Indifference [1] claiming *“Institutional discrimination* [exists] *within the NHS against patients with learning disability”* (p18). This triggered an Independent Inquiry into access to healthcare for people with a learning disability (LD), revealing *“examples of discrimination, abuse and neglect”* [2] (p7)*.* Learning disability, also referred to as intellectual disability, is a disorder with onset during the developmental period that includes both intellectual and adaptive functioning deficits in conceptual, social, and practical domains. The call for urgent action that followed the Inquiry was accompanied by ten essential recommendations for change across the entire healthcare system, including the introduction of LD liaison nurses in hospitals and flagging systems to identify patients with LD [2]. As yet, attention has been placed primarily on the inequalities faced by adult patients with LD [3, 4], with little consideration given to understanding at what point in the patient journey these inequalities occur. Is it, for example, the moment a young person with LD is transferred to adult services or sometime earlier? Consideration of their circumstances suggests that children and young people (CYP) with LD are a vulnerable population who face a greater risk of abuse and accidental injury than CYP without LD: due to factors such as their receipt of intimate, personal care from multiple caregivers; restricted ability to communicate; and impaired capacity to identify or avoid danger [5]. Parents of CYP with LD have reported concerns about the quality, safety and accessibility of hospital care for their child [6–10]. Underpinning many of their concerns are issues around communication between staff and the child with LD, communication between staff and themselves (parents), as well as communication between staff members about the CYP with LD. A recent ethnographic study [9] in England highlighted the importance of *“hospital staff understanding the enormity of emotional and physical costs to CYP with LD … that can arise when the little things that are important to these patients are unrecognised or overlooked”*. Key to delivering these ‘little things’ was being able to identify CYP with LD to find out what reasonable adjustments were needed and for that information to be communicated effectively to whoever needed to know.

All too often hospital staff rely on parents to provide information about their child [9] who can feel a *“weight of responsibility”* about their child’s communication [8] (p747) and overall safety [5] that impacts on their willingness to leave them alone in hospital. The use of hospital passports to document information about the individual needs of patients with LD is thought to be one way of helping to *“overcome difficulties in communication and ensure that appropriate and individualised care is delivered”* (p4), although in the adult setting there is limited evidence of use and effectiveness in practice [11].

In relation to staff, it has been suggested by Sharkey et al. ([8], p748) that their “*lack of confidence or failure to recognize the centrality of communication to health care … may be leading to their failure to make the most effective use of the opportunities that do exist*”. In a recent national mixed methods study of adult LD services, Tuffrey-Wijne et al. [3, 12] highlighted that, “*simply flagging the patient is not sufficient; patients need to be identified by staff as having LD”* (p43). Their review of the factors affecting the implementation of strategies to promote a safer environment for adults with LD in NHS (National Health Service) hospitals [12] found that hospitals with a LD nurse were better able to provide safe and good-quality healthcare for patients with LD than those without, but their effectiveness was limited by inadequate structures of management support and their role was at risk of being marginalised. As Tuffrey-Wijne [3, 12] notes, *“one learning disability liaison nurse clearly cannot achieve organisational change in isolation. Therefore, senior management support for the role has to be embedded within the hospital structures. This includes ensuring that there is sufficient cover and that the role carries sufficient authority and seniority”* (p88).

The Care Quality Commission’s (CQC) view of best practice for the care of CYP with LD includes children’s units having access to senior LD nurse provision, a clearly visible system for flagging these patients when they are admitted to hospital, the use of hospital passports and defined communication strategies [13]. Without a comprehensive evaluation of how well children’s hospital services meet the needs of all CYP, we do not know whether parental concerns for CYP with LD and a long-term condition (LTC) differ from parents of other children with LTC. Moreover, what also remains unclear is the extent to which the CQC recommendations *are* being applied and if so, what *difference* they are making. Ultimately, it is essential that researchers and healthcare professionals working within child hospital settings learn from the costly mistakes that have occurred in the adult hospital setting [1, 2] and minimise the risk of CYP with LD and their families from experiencing the same poor outcomes.

### Research design and methodology

**Fig. 1:**
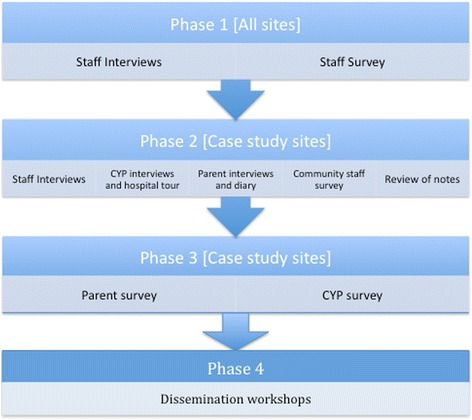
Four phase study design

The Pay More Attention study aims to identify the factors that facilitate and prevent CYP with LD and long term conditions from receiving equal access to high quality hospital care and services, and to compare CYP with and without LD [14]. This is a four-phase study utilising a transformative, mixed methods case study design [15] (Fig. 1).

This paper draws on Phase 1, the aims of which were to: a) describe the organisational context for healthcare delivery to CYP with LD and their families (hospital staff interviews); and b) compare staff perceptions of their ability to identify the needs of CYP with and without LD and their families and provide high quality care to effectively meet these needs (hospital staff survey).

From the outset parents have been involved in all aspects of the study design, including one parent with LD children being a co-investigator. Our patient and public involvement strategy includes a parent advisory group, study steering committee and partnerships with special needs schools for CYP.

### Hypotheses

1. Staff will perceive that they are less able to meet the needs of CYP with LD in terms of their confidence, capability, and capacity, than the needs of CYP without LD.
2. Staff will perceive that CYP with LD and their parents have less involvement in decisions and planning services than CYP without LD.
3. Staff will perceive that CYP with LD are less safe in hospital than CYP without LD.
4. Staff will perceive that CYP with LD are valued less and treated with less dignity and respect than CYP without LD.

### Sample and setting

All designated children’s hospitals in England (*n* = 15) were invited to take part via an email sent to the Trust’s Chief Nurse (or nominated other) through the Association of Chief Children’s Nurses (ACCN) [16]. To recruit non-children’s hospitals, a sampling strategy was developed based on their proximity and referral patterns to the specialist children’s hospitals. Staff in a senior clinical or managerial role or those employed specifically to work with CYP with LD were eligible to take part in interviews. All clinical and non-clinical hospital staff with contact with CYP and their families were eligible to take part in the survey. A local collaborator was identified at each site to facilitate this study.

## Methods

### Staff interviews

Local collaborators identified eligible staff with the relevant experience and expertise to answer the questions, and provided them with an information sheet and consent form. Staff were given the option of conducting the interview face to face or over the telephone. Verbal or written consent was taken at the start of each interview. Interviews were semi-structured, with an interview schedule (Additional file 1) used to direct discussion, focussing on the delivery of services to CYP with LD at the organisational level, including the policies, systems and practices in place to support their care and treatment. Interviews were recorded and transcribed verbatim in all but one instance where extensive notes were taken. A minimum of two interviews per site was planned.

### Staff survey

An anonymised staff survey (Additional file 2) was developed on the basis of existing literature [5–9, 12] in consultation with experts in the field, including parental input. The survey was divided into two parts, part 1 focussing on CYP with long term conditions *with* LD and part 2 focussing on CYP with LTC *without* LD. Definitions of LD and long term condition were provided for clarification. Staff were not asked to distinguish between mild, moderate, severe or profound LD. The survey covered four key areas with the majority of questions being scored on a 5-point Likert scale. Demographic questions ascertained information about the participants. The survey was piloted to check for relevance and acceptability. Survey characteristics are provided in Table 1.

**Table 1:** Survey characteristics

Number of questions	Part 1 Demographics (Gender, Role, Number of years worked in the Hospital, Pay Band)
	Part 2 CYP with LTC *and* LD: 30 questions
	Part 3 CYP with LTC *without* LD: 26 questions
Definitions provided	Long term condition (LTC): Health conditions that last a year or longer, impact on a person’s life, and may require on-going care and support e.g. epilepsy, Cerebral Palsy or diabetes
	Learning Disability (LD): A reduced intellectual ability and difficulty with everyday activities, which affects a person for their whole life
Key areas	Identifying and tracking (LD Only)
	Meeting needs: confidence, capability, capacity
	Equality of access
	Safety
	Values
Primary response format	5 point Likert scale (Strongly agree – Strongly disagree)
Piloting	Seven staff from different professional backgrounds to check relevance and acceptability

The staff interviews and survey were conducted concurrently over a period of 5 months.

### Data analysis

#### Interview data

Staff interviews were analysed thematically on NVIVO 11 using the five stages of the Framework: familiarisation; identifying a thematic framework; indexing; charting; mapping and interpretation [17]. Framework was appropriate for the research as it involves a large amount of data from multiple sites, facilitating analysis within and between sites [17–19].

#### Survey data

Descriptive statistics were used to characterise the sample. Questions about involvement in service delivery and planning services, safety, values and meeting needs were analysed using the Wilcoxon sign-rank test, comparing responses about CYP with LD to those about CYP without LD for the total sample and separately for respondents from children’s and non-children’s hospitals. A Bonferroni correction for multi-comparisons was made, resulting in an alpha level of 0.005. All data were analysed using SPSS version 22.

## Results

The sample of 24 hospitals in England included all 15 specialist children’s hospitals and nine non-children’s hospitals, including district general hospitals and teaching hospitals. One non-children’s hospital declined to take part and two did not respond. Hospitals in urban and rural locations were included. The final sample of participants included 65 staff from 22 hospitals who participated in interviews and 2261 staff from 24 hospitals who completed the survey. A wide range of staff participated including those from various professional backgrounds who had been in their Trust from less than 1 year to over 25 years. Recruitment figures are shown in Table 2. All but three interviews were conducted via the telephone lasting 30-45 min.

**Table 2:** Study participants

Method	Number of Hospital Sites		Number of participants		Staff groups					
					Dr	N	AHP	LDN	SM	Other
Interviews	22	Children’s *n* = 15	65	Children’s *n* = 48	6	15	2	8	13	4
		Non-children’s *n* = 7		Non-children’s *n* = 17	4	11	0	2	1	0
					Dr	N	AHP	HCA	Non-clinical/ other	
Survey	24	Children’s *n* = 15	2261	Children’s *n* = 1681	272	762	308	79	260	
				Range per site 38-202						
		Non-children’s *n* = 9		Non-children’s *n* = 580	105	222	67	50	136	
				Range per site 7-131						

*Dr* Doctor, *N* Nurse, *AHP* Allied health professional, *LDN* Learning Disability Nurse, *SM* Senior manager, *HCA* Healthcare Assistant

Qualitative data analysis revealed two key themes, national variation and staff uncertainty. Staff interviewed from 15 hospitals demonstrated a lack of knowledge or consensus about one or more of the policies, systems and practices in place at the organisation level to support the care of CYP with LD (Table 3). These included whether there was a standalone LD policy, a system for flagging CYP with LD and alerting staff, practices for eliciting feedback from CYP with LD and practices for involving them in planning services.

**Table 3:**
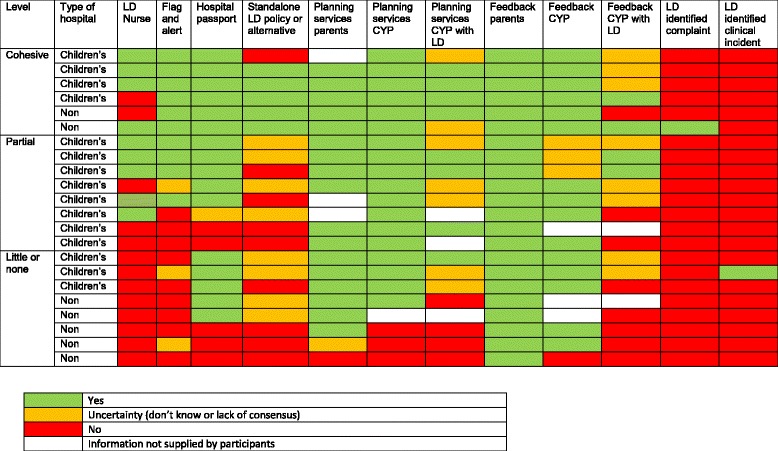
Overview of participant sites

Nationally, there was considerable variation between hospitals ranging from those appearing to have few or no systems, policies or practices in place *specifically* for CYP with LD (three children’s and five non-children’s hospitals), those with partial systems, policies or practices in place (eight children’s hospitals) and those with a cohesive and comprehensive level of provision (four children’s and two non-children’s hospitals). Variation *between* sites and uncertainty between staff *within* sites is reported in further detail in the next section.

### Policies

None of the interviewees at the 22 sites reported having a standalone LD policy for CYP. More commonly, issues related to the care of CYP with LD were integrated into a range of policies (three hospitals) and/or other documents (five hospitals) such as a LD care pathway, protocol, Mencap Charter [20] or standards. The LD care pathway was seen by interviewees to offer a way of prompting staff to *“think about what they need to do”* to make necessary reasonable adjustments for CYP with LD, for example, in relation to the length and timing of out-patients appointments. A LD protocol was described as more prescriptive and different to a pathway in that it was for *“staff to follow”* and about *“making sure that they make reasonable adjustments and giving them the tools to do so”.* One children’s hospital indicated a preference for the use of standards over a LD policy:

“We changed it from a policy to a standard so we could audit against it … because sometimes policies were there to refer to but the standard was trying to make it happen”

Interviewees from nine sites (6 children’s and 3 non-children’s hospitals) reported not knowing what policies, if any, existed or there was a lack of consensus.

### Systems

#### Identifying and tracking CYP with LD

In ten of the 22 hospitals (45%; 8 children’s/2 non-children’s) it was reported during interviews that a flagging and/or alerting system was in place for identifying CYP with a LD, although at two sites there was a lack of clarity about how the system worked in practice. Flagging in all ten sites was electronic; two sites reported a child’s LD was additionally documented in the paper notes and on their hospital passport. The systems for alerting staff of a child’s LD varied. Most commonly, an email was sent to the LD nurse/team within the hospital once a flag was put in place; this could trigger a telephone call by the LD nurse to the ward to ask about reasonable adjustments and/or providing a hospital passport. In one site, a children’s hospital, it was reported that the email notification went to multiple staff including the ‘music therapist’ and ‘disability teacher’, whilst in another a more ad hoc system of sharing information about the child’s needs was place:

“We will put a flag so that when they’re admitted it’s flagged up prior to their admission that they’ve got a learning disability … that’s the main way, on the wards, that they would know that somebody’s come in … when children are planned to come in, **quite often** that information is given to the wards … put in the diary about what they need. If I’ve got children with specific needs, such as they need a special bed with high sides … the consultant would flag that, **quite often** as part of the pre-assessment”*.*

Just under half (*n* = 998) of survey respondents agreed that they were routinely informed of a CYP’s LD. Results were similar for respondents in children’s and non-children’s hospitals (49% and 46% respectively). Five hundred and sixty (24.9%) reported not knowing what systems were in place in their hospital for identifying patients with LD, including 19% of nurses and 28% of doctors. Medical and nursing notes were the most commonly reported systems and a sticker on the notes the least common. Examining quantitative data within sites revealed a lack of agreement about what systems were in place. In one specialist children’s hospital, for example, 62% of staff reported no electronic recording in place, whereas 38% of participants reported the opposite.

#### Complaints and clinical incidents

Interviewees from two sites (one children’s and one non-children’s hospital) reported having a specific mechanism in place for identifying that a CYP at the centre of a complaint or clinical incident had LD, such as a tick box or specific section on the complaints form. A range of practices within and across sites was described, that *could* result in a child’s LD becoming apparent:

“Well you should be able to … hopefully someone will have documented within the incident that that had been the case, and also if you looked up that child, or that patient … then a flag should come up saying they have a learning disability”*.*

Other examples of mechanisms that depended on individual responses to the incident or complaint included the staff knowing the child or identifying LD based on knowledge of the services the child uses. Participants from two hospitals, one children’s and one non-children’s, said that complaints relating to CYP with LD were sent to LD staff, although no specific mechanism was described for identifying that the CYP at the centre of the complaint had a LD.

When interviewed staff were asked about safety in relation to the care of CYP with and without LD, those from 12 sites (9 children’s and 3 non-children’s hospitals) felt there were no differences in their safety concerns and/or the way safety is managed between the two groups, with some suggesting that CYP with LD had the same safety issues as young CYP without LD. Those who reported differences in safety issues between the two groups identified six key areas of risk specific to the care of CYP with LD (Table 4), most of which were associated with the individual CYP.

**Table 4:** Safety issues relevant to the care of children and young people with learning disabilities

Theme	Sub themes
CYP	Challenging behaviour
	Difficulties maintaining personal safety and/or reporting abuse
	Communication impairment
	Physical impairment - risks with moving and handling
	Feeding - risk of choking
	Need for routine/familiarity
	Complexity of care/co-morbidities/medication regimes
	Lack of understanding
Equipment	Lack of appropriate equipment
Staff	Reliance on parents
	Lack of familiarity with moving and handling
	Lack of time
Environment	Inappropriate space
Other people	May pose a risk to physical safety of CYP with LD
Information	Lack of hospital passport
	Lack of information sharing
	Insufficient risk assessment

Survey respondents reported feeling less confident about managing challenging behaviour (*Z* = 23.3; *p* < .001) and always delivering safe care to CYP with LD (*Z* = 21.89; *p* = <.001), compared to CYP without LD. They also felt the environment was less safe for meeting the needs of CYP with LD compared to those without (*Z* = 24.02; *p* < .001). These findings were true for respondents from both children’s and non-children’s hospitals. An interviewee at one children’s hospital reported having a disability risk assessment in place for nursing staff to record reasonable adjustments that should be made for a child on admission.

### Practices

#### Learning disability nurse provision

Interviews revealed that Learning Disability nurse provision was in place in eight (53%) children’s hospitals and one (14%) non-children’s hospital. Provision ranged from 1 to 4 staff per site, working full or part-time in various roles including: lead nurse; liaison nurse; nurse speciality; disability assistant; and nurse consultant. Some LD nurses worked across multiple hospitals, across a hospital and community setting or within children’s adolescent and mental health services only.

#### Identifying and meeting the needs of CYP

Staff were asked in interviews about specific adjustments made for CYP with LD in their hospital. The most frequently reported adjustments were made in relation to communicating with this group of patients. Other adjustments related to the admission process, out-patient appointments, theatre experience, the physical environment, accessing specialist staff, community liaison, sharing information (hospital passport) and supporting parents.

Interviewees from 12 children’s and 4 non-children’s hospitals reported having a hospital passport or equivalent available for the documentation of information about the needs of CYP with LD and communicating these to staff. This tended to be completed at a pre-admission appointment or during a hospital admission. One participant at a children’s hospital highlighted an increase in the number of children coming into hospital with hospital passports, whilst a participant at a non-children’s hospital said families always forget to bring them in. In another non-children’s hospital a lack of *“organisational commitment”* was felt to have prevented the hospital passport being implemented into practice:

“We were working with a group which included parents … to try and develop a passport for children with complex needs. It went quite well but really hasn’t, kind of, been used very much … I don’t think, real, kind of, organisational commitment to it”*.*

Survey questions related to meeting the needs of CYP specifically focused on capacity, capability and confidence. Staff reported less capacity and lower levels of capability and confidence in meeting the needs of CYP with LD compared with those without LD (Capacity: Z = 28.457; *p* < .001; Capability: *Z* = 25.860; *p* < .001; Confidence: *Z* = 28.333; *p* < .001), and this was true for respondents from both children’s and non-children’s hospitals.

#### Provision of information to parents/children and young people

Interviewees reported information for parents tended to be in the form of written information leaflets related to their child’s health condition. Other examples are listed in Table 5. Staff from eight sites (six children’s hospitals/two non-children’s hospitals) described having access to materials aimed specifically at CYP with LD (Table 5). Three sites (2 children’s and 1 non-children’s hospitals) lacked consensus about the type of information provided to families.

**Table 5:** Examples of methods of information exchange between staff and families

Target group	Purpose	Methods used
Parents	Information giving	Condition specific information leaflets
		Admission pack
		Ward book
		Partnership in care nursing document
		Guidelines for parents of children with LD
	Eliciting feedback	Friends and family test
	Planning services	Individual interviews
		Parent groups and forums
		Staff interview panel
		Family days
		Listening weeks
CYP	Eliciting feedback	Friends and family test
		Paper based surveys - word/pictures/symbols
		Verbal or written responses
		Film
		Photography
		Texting
		Sing and sign
	Planning services	CYP groups/forums
		15-step programme: go into a clinical area and within 15 steps look at what you see and how you feel about it
		Spoonful of sugar scheme: nurse meets young person before a consultation to help them prepare questions which they may want to ask
CYP with LD	Information giving	Easy read materials
		Symbols
		Photographs
		Pictures, including picture exchange communication (PECS)

#### Eliciting feedback from parents and children and young people

During staff interviews, a wide array of methods were described to elicit feedback from CYP and their parents (Table 5). Overall there was uncertainty about whether specific adjustments for eliciting feedback from CYP with LD existed within sites. Some staff said that CYP with LD could be given materials aimed at younger children or that staff would help them complete feedback forms.

#### Involvement parents and children and young people in planning services

Two-thirds of staff that were interviewed from children’s hospitals and one-third from non-children’s hospitals described mechanisms for involving parents in planning services (Table 5). These mechanisms tended to be condition/speciality specific. Few participants specifically mentioned how they involved parents of CYP with LD in service delivery. At the majority of sites, CYP were reported to be involved in service delivery primarily through participation in different groups and forums but also included were initiatives such as a the ‘15-step programme’ and ‘a spoonful of sugar scheme’ (Table 5). The question about CYP with LD involvement in planning services was more difficult for interview respondents to answer. Hence, it is much less clear whether or how CYP with LD are involved.

Staff who took part in the survey were asked whether they agreed or disagreed that CYP and parents were routinely involved in making decisions about care and treatment and planning services. Staff thought that CYP with LD were significantly less involved than CYP without LD in decisions about their care (*Z* = 18.225, *p* < .001) and in planning services (*z* = 16.212, *p* < .001) and this was the case for respondents from both children’s and non-children’s hospitals. Staff from children’s hospitals thought that parents of CYP with LD were significantly less involved than parents of CYP without LD in decisions about care (*Z* = 4.606, *p* < .001) and planning services (*Z* = 8.463, *p* < .001), whereas those in non-children’s hospitals did not report any differences with regards to parental involvement in decisions about care (*Z* = 1.127; *p* = .260). Staff in non-children’s hospitals did, however, report that parents of children with LD were less involved in planning services than parents of children without LD (*Z* = 2.893, *p* = .004).

### Values

Staff who completed the survey were asked whether their hospital valued CYP with and without LD, and within their hospital whether CYP with and without LD are always treated with dignity and respect. For both groups of patients, staff were also asked how likely they would be to recommend their hospital to a friend/family member and recommend it as being a good place to work. Scores for a composite variable comprising each of these elements were significantly more negative in relation to CYP with LD than CYP without LD (*Z* = 15.576, *p* < .001). This finding was true for respondents from both children’s and non-children’s hospitals.

### Barriers and facilitators

Staff were asked in interviews to identify barriers and facilitators to CYP with LD gaining access to investigations, procedures and treatments (Table 6). Six areas were perceived to have an impact on access, two of which were family related (CYP and parents) and four hospital related (staff, services, environment and resources/equipment). The overriding message from this data is that CYP with LD need access to: a) staff with LD knowledge and training who in turn can access specialist staff when needed; b) appropriate equipment and resources; and c) appropriate spaces. Additionally, the ability of parents to advocate on behalf of their child should be considered.

**Table 6:** Staff perceptions of the barriers and facilitators to children and young people with learning disability gaining access to investigations, procedures and treatments

	Barriers	Facilitators
Staff	Lack of knowledge about needs of CYP, how to identify CYP	Knowledge of specific needs of CYP
	Lack of access to specialist staff	Access to LD nurses, named paediatricians, play specialists
	Lack of time – plan, meet needs	Preparation and planning
	Lack of training	Access to LD specific training and information
	Lack of power	
	Negative attitudes – not wanting to care for CYP, believing it is parents’ responsibility to provide care, CYP will disrupt other patients without LD	Trust recognition of need to focus on LD and staff ‘champions’
	Reliance on parents	
Environment	Lack of appropriate space – lack of cubicles, too cramped	Access to appropriate space: cubicles, wet room, sensory room
	Lack of quiet space	Access to quiet space
	Lack of wheelchair access	
Service related	Lack of coordination between hospital services and between hospital and community	Streamlining/coordinating appointments and providing flexible services
	Lack of specialist treatments and/or procedures	
	Lack of staff capacity	
	Cost – staffing	
	Waiting times	
	Disparity of care and services – within and across hospitals	
CYP	Unable to cope with delays and disruption to routine	
	Anxiety	
	Feeling stigmatised	
Parents	Lack of knowledge of what is available	
	Lack of ability to articulate child’s needs	
	Too embarrassed to ask for what they need	
	Having an LD themselves	
	Language barrier	
	Feeling overwhelmed or negative about what can be done	
	Do not bring in hospital passport	
		Listening to parents
		Working in partnership with parents
Resources and equipment	Lack of communication tools	Access to communication tools and hospital passport
	Lack of hoists	Access to hoists
	Lack of beds	Access to specialists beds
	Lack of bespoke equipment	Access to adapted eating equipment, developmentally appropriate toys

## Discussion

This paper has set out the organisational context for the care of CYP with LD in 24 English hospitals and compared staff perceptions about their ability to identify and meet the needs of CYP with and without LD. These findings highlight national variation in the many systems, policies, and practices which try to meet the needs of CYP with LD, ranging from hospitals appearing to have little or nothing in place specifically aimed at this group of patients through to those with a cohesive and comprehensive approach to meeting their needs. This variation is seen most clearly in relation to the recommendation for all hospitals to appoint a LD liaison nurse [13], met by only eight of the 15 children’s hospitals in England, with considerable variation in the number of LD nurses they employ, their working hours and tenure, remit, job title and pay. This is despite the greater vulnerability of CYP with LD compared to those without LD [5], their higher rates of hospital admission and evidence from adult services of the positive impact LD nurses can have on the provision of safe and good-quality hospital care for patients with LD [12]. Such disparity in provision reflects an urgent need for greater evidence of the impact of the role of LD nurses to inform workforce planning. Otherwise there is a risk, as found in adult services, that the role will lack the capacity and credibility to be effective [12].

The lack of uniformity across children’s and non-children’s hospitals seems to be accompanied by a culture of uncertainty surrounding what is, and what should, be in place for CYP with LD. Such uncertainty is concerning given that interviews were conducted with senior staff identified locally as being the most appropriate to answer the questions. If staff are not confident about whether a standalone LD policy exists in their hospital, for example, how cognisant can they be about its content and how to operationalise it? It is important to consider to what extent uncertainty within organisations and a lack of common standards across organisations accounts for the concerns parents have about the quality, safety and accessibility of hospital care for CYP with LD, particularly given the heightened sense of risk consciousness they have about their child’s health and well-being [5].

A consistent thread running through these findings was the issue of communication, with a lack of defined strategy [13] at the level of the organisation, staff and family. At the organisational level, there was a lack of standardised systems in place for communicating that a CYP in the hospital has a LD. In the ten sites that did have a flagging and alerting system, there was a tendency to rely on the LD nurse to act as the conduit between a CYP being flagged as having LD and staff being alerted of this. With most hospitals having little or no LD nurse provision this raises the issue of how other staff, who are not LD trained, become informed and empowered to identify and meet the needs of these patients. As Tuffrey-Wijne ([3, 12], p43) found “*simply flagging the patient is not sufficient; patients need to be identified by staff as having LD”.* For this they need to have knowledge about LD but also knowledge of the systems in place within their hospital to identify CYP with LD - something we found was not always the case.

There was also a distinct lack of systems in place for recording that a CYP involved in a complaint or the subject of clinical incident has LD, with reliance instead on individual staff to recognise a child’s name and that they have LD and communicate this information. Our hypothesis, that staff will perceive that CYP with LD are less safe in hospital that CYP without LD, was supported. This, together with the wider context of patients with LD being at increased risk of preventable death [21] as well as injury [22] and abuse [23, 24] represents a lost opportunity to identify relevant risk factors and preventative mechanisms. However, not all staff at the senior level recognised a difference in safety concerns between CYP with and without LD. A disability risk assessment for recording necessary reasonable adjustments, used in one children’s hospital, may be one way of minimising risk and also alleviating parental concerns such that they feel able to leave their child alone in hospital [5, 8].

At the staff level, hospital passports are repositories of key information about CYP with LD, which if communicated to and between staff should, over time, improve their knowledge and understanding. Despite the use of hospital passports being viewed by the CQC as best practice for the care of CYP with LD, these were not universally in place across hospitals in our study. What we also do not know is how successfully they are being used, with some sites reporting increased use by parents and others indicating that parents do not bring them with them into hospital.

There is a clear need for further work at the family level in relation to staff communication and engagement. Despite a number of innovative ways being used by staff in some hospitals to inform, engage and communicate with CYP, it was apparent that these were not routinely adapted for CYP with LD. Moreover, our hypothesis that staff will perceive that CYP with LD have less involvement in decisions and planning services than CYP without LD was supported. Such inequality needs to be addressed through investment in appropriate training at both the organisation and individual level, the provision of appropriate resources and robust evaluation and dissemination. Consideration also needs to be given as to why parents of CYP with LD are viewed by staff as being less involved than parents of CYP without LD; given the increased need for the former to advocate for their child, and because they may have much more contact with hospital services overall [25].

Children and young people with LD often face additional challenges communicating their needs and wishes or reporting pain and discomfort, fears or abuse [22]. It is therefore vital that systems, policies and practices in place to help safeguard them are joined up and underpinned by a robust strategy for ensuring good communication at every level. Our hypothesis that staff feel less able to meet the needs of CYP with LD in terms of their confidence, capability, capacity, than the needs of CYP without LD, indicate that current provision is insufficient. This could account for why staff also perceived that CYP with LD are valued less and treated with less dignity and respect than CYP without LD (supporting our fourth hypothesis).

### Strengths and limitations

The findings reported here are based on 65 interviews and 2261 surveys from staff within a range of hospitals across England. Triangulation of the interview and survey data strengthens the study findings. Interviews were predominantly conducted over the telephone and designed to last approximately 30 min to accommodate participants’ work demands, which means we cannot say with certainty that our organisational mapping is complete. Whilst we collected over 2000 survey responses, our sampling method meant that we were not able to determine a meaningful response rate in terms of representativeness of different professional groups. Furthermore, participants were self-selecting, which means the findings cannot be generalised with any degree of precision. In all participating sites we were reliant on the local collaborator to distribute the survey to staff that had contact with CYP with and without LD. It is possible that in non-children’s hospitals not all eligible staff were identified and given the opportunity to participate. Similarly, we were dependent on the local collaborator to identify senior staff with the knowledge to answer the interview questions and it is possible that we did not include those most informed within the organisation despite trying to address this limitation with at least two interviews per site.

## Conclusion

The first national study of the equality of hospital care for CYP with LTC has revealed significant disparities between those with and without LD. Staff from both children’s and non-children’s hospitals perceived that those with LD were included less, valued less, and less safe than CYP without LD. They also reported having less confidence, capability and capacity to meet the needs of this population compared to those without LD.

The nature and extent of organisational policies, systems and practices in place within hospitals to support the care of CYP with LD differs across England and some uncertainty exists within and across hospitals as to what is currently available and accessed. Findings indicate inequality with regards the provision of high quality hospital care to children and young people with LD that meets their needs. There is a pressing need to understand the impact this has on them and their families.

## Additional files

Additional file 1: Staff Interview Schedule. (DOCX 17 kb)

Additional file 2: Staff Survey. (PDF 987 kb)

## Abbreviations

ACCN: Association of chief children’s nurses
CQC: Care quality commission
CYP: Children and young people
LD: Learning disability
LTC: Long-term conditions
NHS: National health service

## References

[1] Mencap. Mencap death by indifference. London: Mencap; 2007.

[2] Michael J (2008). Healthcare for all: report of the independent inquiry into access to healthcare for people with learning disabilities.

[3] Tuffrey-Wijne I, Giatras N, Goulding L, Abraham E, Fenwick L, Edwards C, Hollins S. Identifying the factors affecting the implementation of strategies to promote a safer environment for patients with learning disabilities in NHS hospitals: a mixed-methods study. NIHR J Libr. 2013. 10.3310/hsdr01130. 25642531

[4] Tuffrey-Wijne I, Goulding L, Giatras N, Abraham E, Gillard S, White S, Edwards C, Hollins S. The barriers to and enablers of providing reasonably adjusted health services to people with intellectual disabilities in acute hospitals: evidence from a mixed-methods study. BMJ Open. 2014. 10.1136/bmjopen-2013-004606. 10.1136/bmjopen-2013-004606 PMC3996812 24740978

[5] Oulton K, Heyman B (2009). Devoted protection: how parents of children with severe learning disabilities manage risk. Health, Risk Soc.

[6] Avis M, Reardon R (2008). Understanding the views of parents of children with special needs about the nursing care their child receives when in hospital: a qualitative study. J Child Health Care.

[7] Brown FJ, Guvenir J (2009). The experiences of children with learning disabilities, their carers and staff during a hospital admission. British J Learn Dis.

[8] Sharkey S, Lloyd C, Tomlinson R, Thomas E, Martin A, Logan S, Morris C. Communicating with disabled children when inpatients: barriers and facilitators identified by parents and professionals in a qualitative study. Health Expect. 2016;19(3):738-50. 10.1111/hex.12254. Epub 2014 Aug 24. 10.1111/hex.12254 PMC5055242 25156078

[9] Oulton K, Sell D, Kerry S, Gibson F. Individualizing hospital care for children and young people with learning disabilities: it's the little things that make the difference. J Pediatr Nurs. 2015. 10.1016/j.pedn.2014.10.006. 10.1016/j.pedn.2014.10.006 25450442

[10] Shilling V, Edwards V, Rogers M, Morris C. The experience of disabled children as inpatients: a structured review and synthesis of qualitative studies reporting the views of children, parents and professionals. Child Care Health Dev. 2012. 10.1111/j.1365-2214.2012.01372.x. 10.1111/j.1365-2214.2012.01372.x 22372968

[11] Sheehan R, Gandesha A, Hassiotis A, Gallagher P, Burnell M, Jones G, Kerr M, Chaplin R, Crawford MJ. An audit of the quality of inpatient care for adults with learning disability in the UK. BMJ Open. 2016. 10.1136/bmjopen-2015-010480. 10.1136/bmjopen-2015-010480 PMC4838729 27091821

[12] Tuffrey-Wijne I, Giatras N, Goulding L, Abraham E, Fenwick L, Edwards C, Hollins S (2013). Identifying the factors affecting the implementation of strategies to promote a safer environment for patients with learning disabilities in NHS hospitals: a mixed methods study. Health Serv Deliv Res.

[13] Glasper A (2017). Optimising the Care of Children with intellectual disabilities in hospital. Compr Child Adolescent Nurs.

[14] Oulton K, Wray J, Carr L, Hassiotis A, Jewitt C, Kerry S, Tuffrey-Wijne I, Gibson F. Pay more attention: a national mixed methods study to identify the barriers and facilitators to ensuring equal access to high-quality hospital care and services for children and young people with and without learning disabilities and their families. BMJ Open. 2016. 10.1136/bmjopen-2016-012333. 10.1136/bmjopen-2016-012333 PMC5168702 27940626

[15] Creswell JW, Plano Clark VL (2011). Designing and conducting mixed methods research 2nd Ed.

[16] Association of Chief Children’s Nurses http://accnuk.org/ Accessed 1 Aug 2017.

[17] Ritchie J, Spencer L, Bryman A, Burgess B (1994). Qualitative data analysis for applied policy research. Analyzing qualitative data.

[18] Gale NK, Heath G, Cameron E, Rashid S, Redwood S. Using the framework method for the analysis of qualitative data in multi-disciplinary health research. BMC Med Res Methodol. 2013. 10.1186/1471-2288-13-117. 10.1186/1471-2288-13-117 PMC3848812 24047204

[19] Smith J, Firth J (2011). Qualitative data analysis: the framework approach. Nurse Res.

[20] Mencap Getting it Right Charter 2012. https://www.mencap.org.uk/search/resources?search=charter&x=0&y=0. Accessed 1 Aug 2017.

[21] Health I, Lives Learning Disabilities Observatory (2012). Confidential inquiry into premature death of people with learning disabilities.

[22] Sherrard J, Tonge BJ, Ozanne-Smith J (2001). Injury in young people with intellectual disability: descriptive epidemiology. Inj Prev.

[23] Miller D, Brown J (2014). We have the right to be safe’ protecting disabled children from abuse.

[24] Spencer N, Devereux E, Wallace A, Sundrum R, Shenoy M, Bacchus C, Logan S (2005). Disabling conditions and registration for child abuse and neglect: a population-based study. Pediatrics.

[25] Mahon M, Kibirige SM (2004). Patterns of admissions for children with special needs to the paediatric assessment unit. Arch Dis Child.

