# Supplementary material for: Mapping staff perspectives towards the delivery of hospital care for children and young people with and without learning disabilities in England: a mixed methods national study
Source: BMC Health Serv Res. 2018 Mar 23;18:203. doi: 10.1186/s12913-018-2970-8 (PMC5865304; doi:10.1186/s12913-018-2970-8)
Supplement: Supplementary file 1 — Staff Interview Schedule. (DOCX 17 kb) [file 12913_2018_2970_MOESM1_ESM.docx]

**Interview Schedule for staff**

**Pay More Attention**

**Ensuring equal access to high quality hospital care and services for children and young people with and without learning disabilities: Phase 1**

The following prompts will act as a guide for the researchers on the issues to cover rather than a list of questions to be asked in sequential order.

**Do children and young people (CYP) with and without learning disabilities (LD) and their families have equal access to high quality hospital care that meets their particular needs?**

Prompts

1. Do you have any dedicated LD Nurses (paediatric/adult) in your Trust?
2. What are the policies, procedures and systems for identifying/flagging patients?
3. What data and information are collected on admission from CYP and their parents/carers?
4. What are the perceived barriers to and facilitators of identifying and collecting this type of information (Q2)?
5. Is there a standalone LD policy – why/why not?

**Do CYP with and without LD, assisted by their families, have equal access to hospital appointments, investigations and treatments?**

Prompts

1. What policies and procedures are in place for monitoring and managing CYP’s in-patient and out-patient activity?
2. What are the perceived barriers and facilitators to CYP gaining access to hospital appointments, investigations and treatments?

**Are CYP with and without LD and their families equally involved as active partners, in their treatment, care and services?**

Prompts

1. Is there a policy for involving CYP and parents/carers as effective partners?
2. What, if any ‘reasonable adjustments’ are made to enable CYP and parents/carers to be involved?
3. Is there a guideline informing the provision of information, practical support and service co-ordination for parent/carers?
4. What policies and procedures are in place to involve CYP and their parents/carers in the planning of services?
5. Are Hospital Passports used?

**Are CYP with and without LD and their families equally satisfied with their hospital experience?**

Prompts

1. How is data about patient and parent satisfaction collected, friends and family test, questionnaire or something else?
2. What, if any, specific adjustments are made for those with LD to be able to report on their experience?

**Are there any safety concerns regarding the care of CYP with and without LD?**

Prompts

1. What are the perceived differences in safety issues between these two groups of patients?
2. What processes are in place for recording/monitoring complaints/clinical incidents?

**If time allows, facilitate the staff member to reflect on their own experience**

1. Thinking about these two populations what do you/your Trust do well, what could you/your Trust do better?
2. What do you think are the perceived barriers and facilitators to meeting the particular needs of patients and their families?

How would you describe the key differences with children and young people with learning disabilities – are they seen as children for whom adjustments are made or seen as a specific population?
