# Supplementary material for: Mapping staff perspectives towards the delivery of hospital care for children and young people with and without learning disabilities in England: a mixed methods national study
Source: BMC Health Serv Res. 2018 Mar 23;18:203. doi: 10.1186/s12913-018-2970-8 (PMC5865304; doi:10.1186/s12913-018-2970-8)
Supplement: Supplementary file 2 — Staff Survey. (PDF 987 kb) [file 12913_2018_2970_MOESM2_ESM.pdf]

1. Pay More Attention - Staff Survey V2 25/01/16 IRAS: 193932

Pay More Attention

Ensuring equal access to high quality hospital care and services for children and young people with and without learning disabilities: Phase 1

We are a team of researchers from the Centre for Outcomes and Experience Research in Children's Health, Illness and Disability (ORCHID) at Great Ormond Street Hospital. We are conducting a national study to compare how hospital care and services are provided to, and experienced by, two groups of children and young people with long-term health conditions - those with learning disabilities and those without learning disabilities.

Definitions
Long term conditions are health conditions that last a year or longer, impact on a person's life, and may require on-going care and support e.g. epilepsy, Cerebral Palsy or diabetes

Learning disabilities mean a person has a reduced intellectual ability and difficulty with everyday activities, which affects them for their whole life.

We are inviting all staff from over 20 hospitals in England who come into contact with children and young people (CYP) aged between 5 and 15 years old, with and without learning disabilities and their families to complete this short survey, which should take 5-10 minutes to complete. We want to give staff the opportunity to share their views about providing care to both groups of patients and their families. We want to use the information we learn from this research to help ensure equality of access to high quality hospital care and services for all children and families in the future. The same questions are being asked for those with learning disabilities and those without learning disabilities.

All information collected will be kept strictly confidential and will be used solely for the purpose of the study. By completing the survey you are consenting to taking part in the study. Only members of the research team will have routine access to the data collected. The results will be published and presented so they can be shared with other healthcare professionals and researchers. All data that is shared will be anonymised so that participants cannot be identified.

Contact details:
Chief Investigator: Dr Kate Oulton, 0207 829 7822, kate.oulton@gosh.nhs.uk

Project Manager: Dr Charlotte Kenten, 0207 405 9200 ext: 1771, charlotte.kenten@gosh.nhs.uk

Research Team: Dr Kate Oulton, Prof Faith Gibson, Dr Jo Wray, Prof Margaret Fletcher, Prof Carey Jewitt, Prof Angela Hassiotis, Dr Irene Tuffrey-Wijne, Ms Sam Kerry, Dr Lucinda Carr. Research Associate: Jess Russell

This study is being funded by the National Institute of Health Research (NIHR).

Thank-you

Acknowledgement and Disclaimer: This project was funded by the National Institute for Health Research Health Services and Delivery Research Programme, project number 14/21/45. The views and opinions expressed therein are those of the authors and do not necessarily reflect those of the Health Services and Delivery Research Programme, NIHR, NHS or the Department of Health.

About You

1. Are you male or female?

- ◯ Male
- ◯ Female

2. What is your current NHS band?

- ◯ 1-4
- ◯ 5-6
- ◯ 7
- ◯ 8 and above
- ◯ Not directly employed by NHS e.g. external company

3. How many years have you worked in this Trust?

Years [ ]

4. What role do you have in the Trust? Please mark one circle

- ◯ Doctor
- ◯ Nurse
- ◯ Administration
- ◯ Porter
- ◯ Health Care Assistant
- ◯ Family Support Worker
- ◯ Chaplain
- ◯ Teacher
- ◯ Other therapist
- ◯ Physiotherapist
- ◯ Occupational Therapist
- ◯ Social Worker
- ◯ Psychologist
- ◯ Dietetics
- ◯ Pharmacist
- ◯ Psychiatrist
- ◯ Family Therapist
- ◯ Play specialist
- ◯ Speech and Language
- ◯ Other role not listed
- ◯ Domestic services

Part 1: Identifying and tracking CYP **with learning disabilities**

All the questions in Part 1 (Q.5-20) relate to children or young people with learning disabilities

Please mark one circle for each question unless instructed otherwise.

5. Has your Trust ever given you information about how to define learning disability?

○ Yes ○ No ○ Don't Know

6. How confident are you about identifying that a child or young person in your care/who you meet has a learning disability?

1 Extremely Confident	2	3	4	5 Not at all confident
○	○	○	○	○

7. How useful do you think it is that children and young people with learning disabilities are:

	1 Extremely useful	2	3	4	5 Not at all useful	Don't know
a) identified at an organisational level (e.g. central database, electronic patient record)?	○	○	○	○	○	○
b) visibly flagged at an organisational level (e.g. sticker on patient notes)?	○	○	○	○	○	○

8. What systems are in place in your Trust for identifying and recording that a child or young person has learning disabilities? Tick all that apply

We do not have any systems	○
I do not know	○
Documented in medical notes	○
Documented in nursing records	○
Documented electronically	○
Sticker on patient notes	○
Database	○

Other (please specify)

9. In your role, how easy is it for you to use these systems to identify that a child or young person has a learning disability?

1 Extremely easy	2	3	4	5 Not at all easy	I do not know
○	○	○	○	○	○

10. In my role I am routinely informed that a child or young person has a learning disability

1 Strongly agree	2	3	4	5 Strongly disagree
○	○	○	○	○

Part 1: Meeting the needs of CYP **with learning disabilities**

11. For children and young people with a long term condition and learning disabilities ...

Please mark one circle for each statement

	1 Strongly agree	2	3	4	5 Strongly disagree
a) I have the necessary knowledge and skills to meet their needs	○	○	○	○	○
b) I have the necessary training to meet their needs	○	○	○	○	○
c) I routinely have access to the necessary resources to meet their needs e.g. communication aids, equipment	○	○	○	○	○
d) I routinely have access to additional specialist support to meet their needs e.g. play specialist	○	○	○	○	○
e) I routinely have access to addtional learning disability specialist staff to meet their needs	○	○	○	○	○
f) I feel confident to communicate effectively with them	○	○	○	○	○
g) I feel confident to assess and manage pain	○	○	○	○	○
h) I feel confident to safely manage challenging behaviour	○	○	○	○	○
i) I work in an environment that is safe for meeting their needs	○	○	○	○	○
j) I am always able to deliver safe care	○	○	○	○	○
k) I work in an environment that is designed to take into account their individual needs	○	○	○	○	○

12.

Please read the following statement about **Reasonable Adjustments:** The law says that all health services must think about people with disabilities. They have to ask 'What extra things do we need to do, so people with disabilities can get health services as good as other people?' These are called Reasonable Adjustments.

Please mark one circle for each statement

	1 Strongly agree	2	3	4	5 Strongly disagree
a) I feel able to identify what reasonable adjustments are needed for children and young people with a long term condition and learning disabilities	○	○	○	○	○
b) I feel confident that any reasonable adjustments will be accommodated in a timely way	○	○	○	○	○

Part 1: Equality of access for CYP with a long term condition **and learning disabilities**

Please mark one circle for each statement

13. I routinely involve children and young people with a long term condition and learning disabilities ...

	1 Strongly agree	2	3	4	5 Strongly disagree
a) when making decisions about their care and treatment	○	○	○	○	○
b) parents in making decisions about their care and treatment	○	○	○	○	○

14. In my Trust children and young people with a long term condition and learning disabilities ...

	1 Strongly agree	2	3	4	5 Strongly disagree	Don't know
a) are routinely involved in the planning of services	○	○	○	○	○	○
b) parents are routinely involved in the planning of services	○	○	○	○	○	○
c) parents are relied upon too much by hospital staff	○	○	○	○	○	○

15.

In my Trust, children and young people with a long term condition and learning disabilities have appropriate access to:

Please mark one circle for each item

	1 Strongly agree	2	3	4	5 Strongly disagree	Not applicable
Medical care and equipment	○	○	○	○	○	○
Educational provision	○	○	○	○	○	○
Play and stimulation	○	○	○	○	○	○
Appointments	○	○	○	○	○	○
Double appointments	○	○	○	○	○	○
First or last clinic appointments	○	○	○	○	○	○
Flexible appointments	○	○	○	○	○	○

Part 1: Overall for children and young people **with learning disabilities**

Please mark one circle for each statement.

16. In my Trust, I feel children and young people with a long term condition and learning disabilities ...

	1 Strongly agree	2	3	4	5 Strongly disagree
a) are always treated with dignity and respect	○	○	○	○	○
b) parents are always treated with dignity and respect	○	○	○	○	○

17. Overall I think my Trust values ...

	1 Strongly agree	2	3	4	5 Strongly disagree
a) children and young people with a long term condition and learning disabilities	○	○	○	○	○
b) parents of children and young people with a long term condition and learning disabilities	○	○	○	○	○

18. How likely would you be to recommend your Trust ...

	1 Extremely likely	2	3	4	5 Not at all likely
a) to a friend or family member of a child or young person with a long term condition and learning disabilities who needed treatment?	○	○	○	○	○
b) as a good place to work with children and young people with a long term condition and learning disabilities?	○	○	○	○	○

19. What could your Trust do to support children better with a long term condition and learning disabilities?

20. What does your Trust do well to support children and young people with a long term condition and learning disabilities?

Part 2: Meeting the needs of CYP with a long term condition **without** learning disabilities

All the questions in Part 2 (Q. 21-30) relate to children and young people without learning disabilities

Please mark one circle for each statement.

21. For children and young people with a long term condition without learning disabilities ....

	1 Strongly agree	2	3	4	5 Strongly disagree
a) I have the necessary knowledge and skills to meet their needs	○	○	○	○	○
b) I have the necessary training to meet their needs	○	○	○	○	○
c) I routinely have access to the necessary resources to meet their needs	○	○	○	○	○
d) I routinely have access to additional specialist support to specifically meet their needs e.g. play specialist	○	○	○	○	○
e) I feel confident to communicate effectively with them	○	○	○	○	○
f) I feel confident to assess and manage pain	○	○	○	○	○
g) I feel confident to safely manage challenging behaviour	○	○	○	○	○
h) I work in an environment that is safe for meeting their needs	○	○	○	○	○
i) I am always able to deliver safe care	○	○	○	○	○
j) I work in an environment that is designed to take into account their individual needs	○	○	○	○	○

22.

Please read the following statement about **Reasonable Adjustments:** The law says that all health services must think about people with disabilities. They have to ask 'What extra things do we need to do, so people with disabilities can get health services as good as other people?' These are called Reasonable Adjustments.

Please mark one circle for each statement

	1 Strongly agree	2	3	4	5 Strongly disagree
a) I feel able to identify what reasonable adjustments are needed for children and young people with a long term condition and without learning disabilities	○	○	○	○	○
b) I feel confident that any reasonable adjustments will be accommodated in a timely way	○	○	○	○	○

Part 2: Equality of access for CYP with a long term condition and **without** learning disabilities

Please mark one circle for each statement

23. I routinely involve children and young people with a long term condition and without learning disabilities ...

	1 Strongly agree	2	3	4	5 Strongly disagree
a) in making decisions about their care and treatment	○	○	○	○	○
b) parents in making decisions about their care and treatment	○	○	○	○	○

24. In my Trust children and young people with a long term condition and without learning disabilities ...

	1 Strongly agree	2	3	4	5 Strongly disagree	Don't know
a) are routinely involved in the planning of services	○	○	○	○	○	○
b) parents are routinely involved in the planning of services	○	○	○	○	○	○
c) parents are relied upon too much by hospital staff	○	○	○	○	○	○

25.

In my Trust, children and young people with long term condition and without learning disabilities have appropriate access to:

Please mark one circle for each item listed

	1 Strongly agree	2	3	4	5 Strongly disagree	6 Not applicable
Medical care and equipment	○	○	○	○	○	○
Educational provision	○	○	○	○	○	○
Play and stimulation	○	○	○	○	○	○
Appointments	○	○	○	○	○	○
Double appointments	○	○	○	○	○	○
First or last clinic appointments	○	○	○	○	○	○
Flexible appointments	○	○	○	○	○	○

Part 2: Overall

26. In my Trust, I feel children and young people with a long term condition and without learning disabilities ...

	1 Strongly agree	2	3	4	5 Strongly disagree
a) are always treated with dignity and respect	○	○	○	○	○
b) parents are always treated with dignity and respect	○	○	○	○	○

27. Overall I think my Trust values ...

	1 Strongly agree	2	3	4	5 Strongly disagree
a) children and young people with a long term condition and without learning disabilities	○	○	○	○	○
b) parents of children and young people with a long term condition and without learning disabilities	○	○	○	○	○

28. How likely would you be to recommend your Trust ...

	1 Extremely likely	2	3	4	5 Not at all likely
a) to a friend or family member of a child or young person with a long term condition and without learning disabilities who needed treatment?	○	○	○	○	○
b) as a good place to work with children and young people with a long term condition and without learning disabilities	○	○	○	○	○

29. What could your Trust do better to support children with a long term condition and without learning disabilities?

30. What does your Trust do well to support children and young people with a long term condition and without learning disabilities?

Thank you for taking the time to complete this survey.
